# Conversion of Natural Aldehydes from *Eucalyptus citriodora, Cymbopogon citratus,* and *Lippia multiflora* into Oximes: GC-MS and FT-IR Analysis †

**DOI:** 10.3390/molecules14093275

**Published:** 2009-08-31

**Authors:** Igor W. Ouédraogo, Michael Boulvin, Robert Flammang, Pascal Gerbaux, Yvonne L. Bonzi-Coulibaly

**Affiliations:** 1 Laboratoire de Chimie Organique: Structure et Réactivité; UFR-SEA, Université de Ouagadougou, 03 BP 7021, Ouagadougou 03, Burkina Faso; 2 Laboratoire de Chimie Organique, Centre de Spectrométrie de Masse, Université de Mons, Place du Parc 20, B-7000 Mons, Belgique; E-mail: pascal.gerbaux@umh.ac.be (P.G.)

**Keywords:** essential oils, aldehydes, GC-MS, oximes, nitriles

## Abstract

Three carbonyl-containing extracts of essential oils from *Eucalyptus citriodora* (Myrtaceae), *Cymbopogon citratus* (Gramineae) and *Lippia multiflora* (Verbenaceae) were used for the preparation of oximes. The reaction mixtures were analyzed by GC-MS and different compounds were identified on the basis of their retention times and mass spectra. We observed quantitative conversion of aldehydes to their corresponding oximes with a purity of 95 to 99%. *E* and *Z* stereoisomers of the oximes were obtained and separated by GC-MS. During GC analysis, the high temperature in the injector was shown to cause partial dehydratation of oximes and the resulting nitriles were readily identified. Based on FT-IR spectroscopy, that revealed the high stability and low volatility of these compounds, the so-obtained oximes could be useful for future biological studies.

## Introduction

Aldehydes are widely used compounds in perfumes and pharmaceutical preparations. The main disadvantage of these molecules is their intrinsic instability and propensity to oxidation. This inconvenience, together with their high volatility, in the case of low molecular weight molecules used in the perfumery field, for instance, makes the use of aldehydes less appealing for some applications [1,2]. In some cases, the corresponding oximes have been proven to present pleasant odour and their easy accessibility from carbonyl compounds was demonstrated. As a consequence, those compounds have been used as olfactory agents in various perfume compositions, instead of the corresponding carbonyl compounds [3,4].

Concerning pharmacology, several studies have shown that oximes present properties as antitumor [5], antimicrobial [6,7], antioxidant [8], anti-depressive [9], anticonvulsant [7], and antiviral agents [10], etc. Many oximes also were investigated in the context of heavy metal complexation [11,12] and gustative [13] properties.

Consequently, finding methods dealing with oxime or oxime acetate preparations from natural molecules or starting directly from crude essential oils is of interest and indeed was already reported, albeit involving subsequent purification steps [14,15].

Recently, some of us demonstrated that natural aldehydes can be extracted in very good yields from essential oils by performing a bisulphite extraction [16]. As an alternative to the standard oximation procedures realized on the crude essential oils [15], we propose in the present work to use the carbonyl extract (CE) rich in aldehydes obtained from essential oils by a bisulphite extraction.

In this paper, we describe preparation of aldoximes from natural aldehydes from *Eucalyptus citriodora*, *Cymbopogon citratus*, and *Lippia multiflora* essential oils in the presence of hydroxylamine hydrochloride. After chemical transformation each product was analyzed without any organic solvent extraction. GC-MS methodology was used to confirm oxime formation. FT-IR spectroscopy was used to check the stability and the volatility of crude oxime products (OP) compared with those of the initial aldehyde extracts.

## Results and Discussion

### Chemical composition of oximation products

As recently reported [16], the studied essential oil aldehydic extracts are constituted by different molecules: citronellal for *Eucalyptus citriodora*; neral and geranial for *Cymbopogon citratus;* and neral, geranial and perialdehyde) for *Lippia multiflora*. These molecules, their expected aldoximes and the nitrile homologues observed in this study, are presented in Table 1.

The oximation products (OP) of the carbonyl extracts (CE) from these three essential oils were obtained as described in the Experimental section. Such a synthetic protocol for oxime preparation is easy to use and thanks to its use of cheap starting materials, represents an economic route. The oxime products (OP), obtained as colorless oils with a nice aromatic smell, were studied by GC-MS and FT-IR spectroscopy analysis.

**Table 1:** Structures of various aldehydes studied and names of oxime and nitrile homologues.

Structures and names				
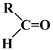	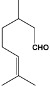	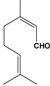	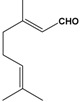	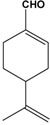
1a-d	1a	1b	1c	1d
	Citronellal	Neral	Geranial	Perillaldehyde
2a-d (*E*)	2a (*E*)	2b (*E*)	2c (*E*)	2d (*E*)
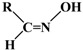	(*E*) Citronellal oxime	(*E*) Neral oxime	(*E*) Geranial oxime	(*E*) Perillaldehyde oxime or (*E*) Perillartine
2a-d (*Z*)	2a (*Z*)	2b (*Z*)	2c (*Z*)	---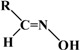	(*Z*) Citronellal oxime	(*Z*) Neral oxime	(*Z*) Geranial oxime	
3a-d	3a	3b	3c	3d
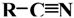	Citronellal nitrile	Neral nitrile	Geranial nitrile	Perillaldehyde nitrile

**Figure 1:**
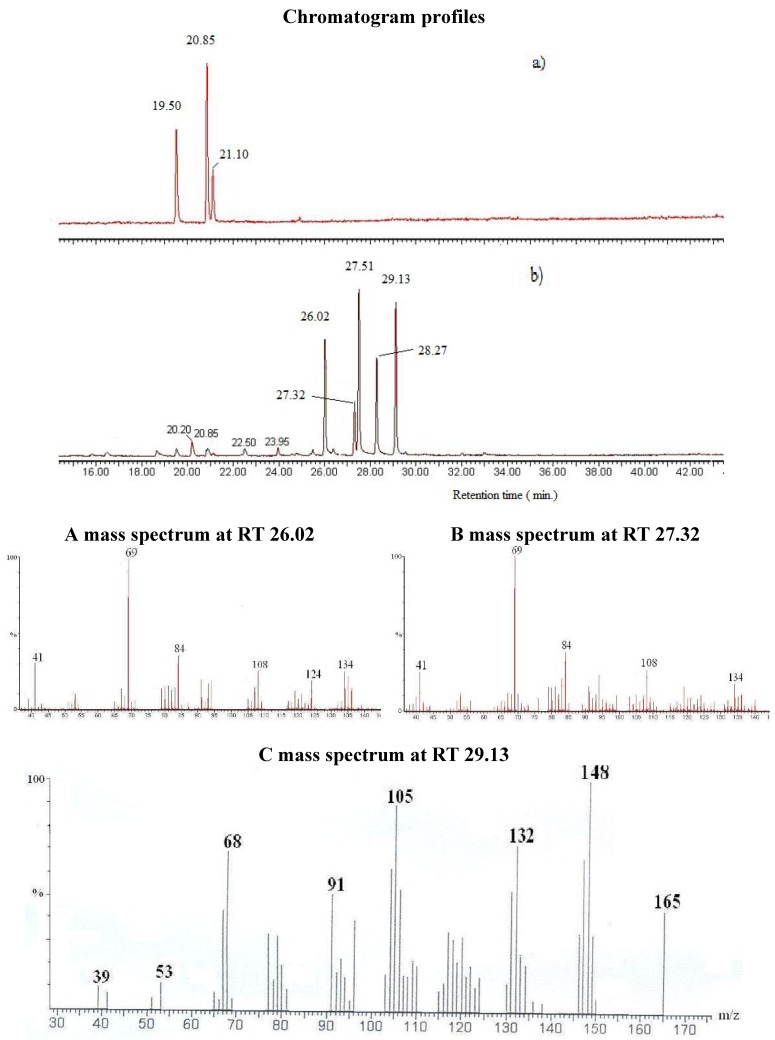
Comparative changes in GC chromatogram profiles of *Lippia multiflora* a) carbonyl extract (CE), b) oximation product (OP) and mass spectra of the oximes. Spectrum A: compound 2b (Isomer I) neral oxime (I), spectrum B: compound 2b (Isomer II) neral oxime (II), spectrum C: compound 3d perialdehyde oxime.

GC-MS analysis of the OP allowed the identification and the quantification of the various components. As representative example, the chromatograms obtained from *Lippia multiflora* carbonyl extract (CE) and its oximation products (OP) are shown in Figure 1. Immediately, we can establish that the chromatographic conditions are optimal since the chromatograms (Figure 1) present very good peak separation that allows the determination of the retention times.

Table 2 shows the various constituents observed in the chromatogram and identified in the three OP from the three carbonyl extracts. For each compound, retention time, mass spectra (70 eV) and % m/m are indicated.

**Figure 2:**
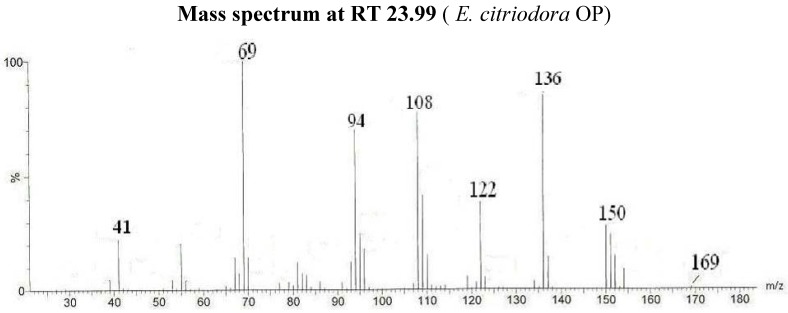
Mass spectrum of compound 2a (Isomer I) citronellal oxime (I).

The identification of the different compounds, either in the CE and the OP, relied on the analysis of the EI mass spectra, which feature signals corresponding to the molecular ions [M^•+^] and to structurally indicative fragment ions. Oximes are characterized by molecular ion peaks detected at *m/z* 169 for citronellal oxime radical cations (Figure 2), at *m/z* 167 for neral oxime (Figure 1) and for geranial oxime radical cations, and at *m/z* 165 for ionized perialdehyde oxime. All spectra exhibit [M-OH^•^]^+^ peaks that are likely to correspond to nitrilium cations. The expected *E* and *Z* geometric isomers that are eluted at different retention times are treated as a sum of the two isomers in Table 2. The comparison of the CE and OP chromatograms in Figure 1 confirms the quasi quantitative conversion of aldehydes to oximes since only traces of unreacted starting carbonyl molecules (citronellal, neral, geranial and perialdehyde) are observed. Indeed, the chromatogram in Figure 1 that corresponds to the GC-MS analysis of OP from *Lippia multiflora* presents five important peaks. Four peaks correspond to the oximes of (i) neral (molecular ion at *m/z* 167) (26.02 min. and 27.32 min.) and of (ii) geranial (molecular ion at *m/z* 167) (27.51 min. and 28.27 min.) as in the case of *C. citratus*. For both oximes, the *E* and *Z* configurations are detected. Obviously, both isomers present the same mass spectra (Figure 1) and are then undistinguishable on the basis of a simple mass spectrometry analyses. 

**Table 2:** Chemical constituents of oximation products (OP) obtained from carbonyl extracts of *E. citriodora, C. citratus, L. multiflora* treated by hydrochloride hydroxylamine.

Compounds	R. T.(min)	m/z ^a^ (70 eV)	Relative concentration (%)^b^					
			*E. citriodora*		*C. citratus*		*L. multiflora*	
			CE.	OP	CE.	OP	CE.	OP
Citronellal	15.70	121/95/136/154/69/111/139/41/55	98.9	0.1				
Neral nitrile	18.67	69/134/148/149/81/121/41/135				1.2		1.6
Citronellal nitrile	18.79	136/69/108/94/122/151/150/41/55		11.7				
Neral	19.52	69/41/94/84/109/119/137/134/123			42.1	--	34.1	0.8
Geranial nitrile	20.20	69/134/148/149/81/121/41/135				3.7		3.4
Geranial	20.85	69/41/84/94/137/123/109/119/134			56.7	0.1	45.1	1.9
Perilaldehyde	21.12	79/68/67/107/135/93/121/150					19.2	0.5
Perilaldehyde nitrile	22.50	105/68/147/104/132/118/67/146						1.8
Citronellal oxime (Isomer I)	23.99	69/136/108/94/122/150/41/55/169		45.3				
Citronellal oxime (Isomer II)	24.86			41.6				
Neral oxime (Isomer I)	26.02	69/150/84/41/94/108/53/167				31.3		23.2
Neral oxime (Isomer II)	27.32					7.3		05.6
Geranial oxime (Isomer I)	27.51	69/84/41/150/108/53/94/167				38.3		26.1
Geranial oxime (Isomer II)	28.27					17.2		14.4
Perillaldehyde oxime	29.13	148/105/132/68/91/165						18.8
**TOTAL**			**98.9**	**98.7**	**98.8**	**99.1**	**98.4**	**98.1**

^a ^Mass spectra data: *m/z* of different peaks are listed following decreasing relative intensities; ^b ^Relative concentration (% m/m) based on GC peak areas without using correction factors.

For open-chain aldoximes, the *E*-isomer, the major and more stable stereoisomer, elutes in general before its corresponding *Z*-isomer. Work is in progress to assign clearly the configuration of each isomer in the three OP, from their analytical and spectral properties: IR,^ 1^H- and ^13^C-NMR as reported for various oximes [17,18,19]. For *L. multiflora* OP, the fifth peak at 29.13 min. (molecular ion at *m/z* 165) corresponds to perilalldehyde oxime, which gives exclusively the *E* isomer as described in the literature for perillartine homologues [20]. The comparison between the chromatograms obtained for the CE analysis and for the OP analysis (see Figure 1) confirms that the oximation procedure affords excellent yields since only small signals are observed that correspond to the starting aldehyde compounds. Table 2 shows that carbonyl compounds remained at 3.2% in the OP of *Lippia multiflora* and 0.1% for the other ones.

Nevertheless, a closer inspection of the OP chromatogram reveals the presence of three weak peaks at 18.65 min., 20.20 min, and 22.50 min. Based on the corresponding mass spectra, it is likely that the corresponding compounds are nitrile molecules that could be generated as a result of thermal dehydration of the oximes during GC analysis. As this point of the discussion, the presence of those nitrile compounds in the starting OP solution can however not be readily ruled out. Actually, vaporization of oximes of neral, geranial and paraldehyde by heating in the injector at 250 °C can be accompanied by dehydration and transformation into the corresponding nitriles prior to the GC separation. The presence of those nitriles in the OP solutions is excluded since no traces of C≡N absorption band (2,260 to 2,210 cm^-1^) were detected in the FT-IR spectrum of OP before GC analysis. For OP from *E. citriodora*, the citronellal-derived nitrile represents the highest signal (11.7% m/m) in the GC analysis, but no band near 2,200 cm^-1^ was observed in the FT-IR spectrum of the corresponding sample. This is a strong indication that nitrile production occurs during the GC analysis. Citronellal oxime, in which the oxime moiety is not conjugated with an ethylenic double bond, seems to undergo the thermal dehydration reaction easier than the neral and geranial oximes.

Data in Table 2 reveal that volatile compounds determined by GC-MS in various extracts (CE and OP) represent 98.1 to 99.1% of the total peak area. The nitrogen-containing compounds observed in the chromatogram represent 94.9% for *Eucalyptus citriodora*, 99% for *Cymbopogon citratus*, and 98.6% for *Lippia multiflora.* This confirms a quasi quantitative transformation of the carbonyl molecules to the corresponding oximes.

When comparing the ratio of geometric isomers genarial /neral that amounts to about 1.3 in CE for *C. citratus* and *L. multiflora* with the OP ratio, geranial oxime/neral oxime about 1.4, we found that there is no significant change and, generally, the used reaction time is sufficient for no chemoselectivity to appear. Quantitative oximation is then observed for each molecule in the case of *L. multiflora* containing three different aldehydes.

Oximes that are polar molecules present, as expected, longer retention times in GC analysis. However, we observed variability in order of retention times for nitriles compared to their corresponding aldehydes. While the retention time of citral-derived nitrile is shorter than that of citral (neral and geranial) retention time, perialdehyde-derived nitrile and citronellal-derived nitrile have retention times longer than those of their corresponding aldehydes. 

### Stability and volatility studies

**Figure 3:**
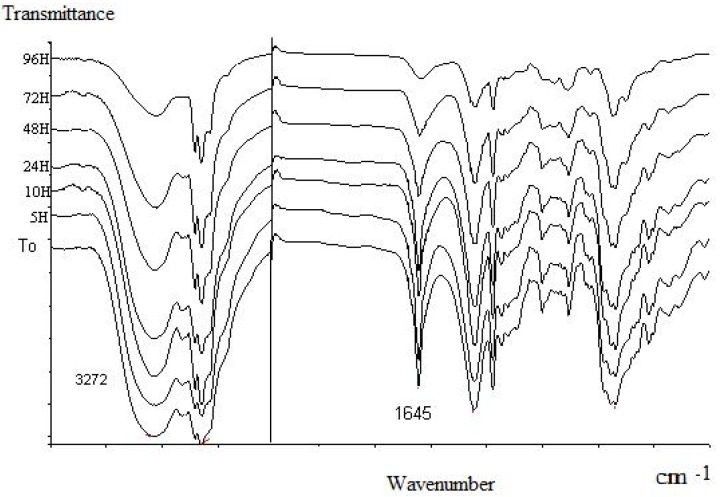
Infra-red spectra of OP from *L. multiflora* essential oil in different times.

We performed infrared (FT-IR) spectroscopy measurements to characterize the oximation products (OP) by the disappearance of νC=O (at 1,676 cm^-1^) and the presence of characteristic broad oxime absorption bands: νO-H near 3,300-3,250 cm^-1^), νC=(N-OH) at 1,645-1,646 cm^-1^ due to the deformation of the C=N. This property was applied to follow also the evolution of oxime oils in time. Volatility and stability studies were performed by preparing KBr pellet charged with oxime product and conserved at room temperature. FT-IR absorption bands on spectra (Figure 3), decreased from time to time. No new bands appeared during the analysis.

Following the ν C=N (at 1,645-1,646 cm^-1^) band, we note that more than 90% of this band reduction was observed just after 96 hours of exposure for *L. multiflora* and for *C. citratus* while the C=O (at 1,676 cm^-1^) band disappears after three hours in the carbonyl extract study (Table 3).

**Table 3:** OP volatility analysis by FT-IR spectroscopy.

Main components in Carbonyl Extract (*Aromatic plant*)	Time* (hour) CE/OP
Citronellal *( E. citriodora )*	3/5
Neral, geranial *(C. citratus)*	5/96
Neral, geranial, perialdehyde *(L. multiflora)*	5/96

* Time corresponding to over 90% reduction of the ν C=O band (at 1676 cm^-1^) or the ν C=N band (at 1,645-1,646 cm^-1^) absorption intensity.

We note that citronellal oxime from *E. citriodora* displays significant important volatility: five hours were enough to reduce the ν C=N (1,654 cm^-1^) band intensity by 90%. This is in agreement with the lowest retention time of this molecule and its facility for dehydration giving a high percentage of nitrile (11.7%) during oxime analysis by GC.

Aldehydes quickly volatilize in the environment and do not persist for longer periods. This drawback limits their biological efficiency. Chemical stability is greater for oxime products (OP) as compared to the corresponding carbonyl extract (CE). The FT-IR spectra show the very slow volatilization of oximes, and this parameter is useful for cosmetic use and it would be highly beneficial for some biological or medical applications.

## Experimental

### Samples and Materials

Aldehydes compounds used in this work for oximation were extracted from commercially available essential oils of *Cymbopogon citratus* (Gramineae), *Lippia multiflora* (Verbenaceae) and *Eucalyptus citriodora* (Myrtaceae) via their bisulphite adducts [16]. Sodium carbonate (Na_2_CO_3_, Acros) solution (10%, m/v) was used for aldehyde regeneration.

### Oxime preparation

A aqueous solution (20 mL) of hydroxylamine hydrochloride (1.2 g, 17.3 mmol) and sodium acetate trihydrate (2.4 g, 17.6 mmol) were added to aldehyde extracts (2 mL) in 95% ethanol (25 mL). The reaction mixture was heated under stirring at 70 °C (60 °C for *Eucalyptus citriodora)* for 1 hour. The mixture was then diluted with distilled water (50 mL) and cooled. The organic phase was removed by decantation, washed with sodium carbonate solution (1%) and with distilled water. The oxime obtained as colourless oil was dried on anhydrous sodium sulphate and stored at -20°C until GC-MS and FT-IR spectroscopy analysis. Experiments were performed twice and oxime yield was calculated as follows: volume of oxime product/starting aldehyde extract volume used (v/v). Experimental values are 0.88 mL/mL for *Eucalyptus citriodora*, 1.0 mL/mL for *Cymbopogon citratus* and 1.1 mL/mL for *Lippia multiflora.*

### Gas chromatography/mass spectrometry analysis (GC-MS)

*Sample preparation and GC-MS analysis:* The GC-MS analyses were performed using a Waters GCT Premier instrument based on an orthogonal injection time-of-flight analyzer. One µL of hexane solution (1/10^5^ v/v) was injected for all the measurements. The gas chromatograph was equipped with a Restek Rtx-5Sil MS column (30 m length, 0.25 mm ID and 0.25 μm DF). Typical GC conditions were: injector temperature, 250 °C; splitless mode; Helium carrier gas flow rate, 1 mL/min; interface temperature: 250 °C. The temperature program was as follow: initial temperature, 55 °C; 1 °C/min ramp; final temperature, 150 °C; 5 °C/min ramp; final temperature, 250 °C (hold 5 min). The relative concentration (%) of the individual component was calculated based on GC peak areas observed without using correction factors. 

Electron Ionization (EI) source conditions were: source temperature, 200 °C; electron energy, 70 eV; trap current, 200 µa; emission current, 400 µa. All ions were transmitted into the pusher region of the time-of-flight analyzer where they were mass analyzed with a 1 s integration time. Data were acquired in continuum mode. The GCT Premier instrument is a high sensitive instrument and, for instance, in EI positive ionization mode, 1 pg of hexachlorobenzene gives S/N > 10/1 whilst acquiring full spectra over a mass range up to *m/z* 800.

*Mass spectra data analysis:* The MS fragmentation pattern was compared with those of pure compound, by matching the MS fragmentation patterns with National Institute of Standards and Technology (NIST) MS Search/version 4.0 mass spectra libraries and with those given in literature.

### Infra-red (IR) analysis

The IR spectra in KBr pellets were recorded using a Perkin Elmer MBX II spectrophotometer. For the volatility study, 0.25 µL of the selected sample was deposited in the middle of a KBr pellet and the IR spectrum was recorded at different times until total evaporation. 

*FT-IR spectra data of oximation products from different essential oils:* IR KBr ν_max_ (cm^-1^): *Eucalyptus citriodora*: 3370, 3255, 2964, 2916, 1654, 1457, 1379, 1348, 1177, 1117, 1045, 927, 822, 741, 696, 606; *Cymbopogon citratus:* 3248, 2916, 1646, 1444, 1377, 1348, 1202, 1105, 939, 856, 818, 619; *Lippia multiflora*: 3369, 3272, 2918, 1645, 1448, 1378, 1349, 1202, 1106, 938, 819, 670, 619.

## Conclusions

The great importance of essential oils has guided our work on chemical transformation of these products to enhance their biological and economic value. Oximes were prepared with three carbonyl extracts from *Cymbopogon citratus* (Gramineae)*, Eucalyptus citriodora* (Myrtaceae) and *Lippia multiflora* (Verbenaceae) essential oils. The carbonyl extracts used have high aldehyde purity due to the extractive method applied before the oximation reaction. The natural aldehydes are converted qualitatively and quantitatively into the corresponding oximes with hydroxylamine hydrochloride.

Oximation products obtained in this study are rich in oximes with purity of 95% to 99%. Very few initial aldehydes remained. According to GC-MS analysis, it is worth mentioning that during heating at high temperature in the GC, oximes underwent dehydration and produced nitriles in low quantities. The oximation method used does not require the use of organic solvents is recommended for amino derivative synthesis. The oxime products formed are more stable and less volatile than the parent aldehydes. This study opens perspectives in biological tests for alimentary protection and cosmetic applications.
